# Fine-Mapping the *HOXB* Region Detects Common Variants Tagging a Rare Coding Allele: Evidence for Synthetic Association in Prostate Cancer

**DOI:** 10.1371/journal.pgen.1004129

**Published:** 2014-02-13

**Authors:** Edward J. Saunders, Tokhir Dadaev, Daniel A. Leongamornlert, Sarah Jugurnauth-Little, Malgorzata Tymrakiewicz, Fredrik Wiklund, Ali Amin Al Olama, Sara Benlloch, David E. Neal, Freddie C. Hamdy, Jenny L. Donovan, Graham G. Giles, Gianluca Severi, Henrik Gronberg, Markus Aly, Christopher A. Haiman, Fredrick Schumacher, Brian E. Henderson, Sara Lindstrom, Peter Kraft, David J. Hunter, Susan Gapstur, Stephen Chanock, Sonja I. Berndt, Demetrius Albanes, Gerald Andriole, Johanna Schleutker, Maren Weischer, Børge G. Nordestgaard, Federico Canzian, Daniele Campa, Elio Riboli, Tim J. Key, Ruth C. Travis, Sue A. Ingles, Esther M. John, Richard B. Hayes, Paul Pharoah, Kay-Tee Khaw, Janet L. Stanford, Elaine A. Ostrander, Lisa B. Signorello, Stephen N. Thibodeau, Daniel Schaid, Christiane Maier, Adam S. Kibel, Cezary Cybulski, Lisa Cannon-Albright, Hermann Brenner, Jong Y. Park, Radka Kaneva, Jyotsna Batra, Judith A. Clements, Manuel R. Teixeira, Jianfeng Xu, Christos Mikropoulos, Chee Goh, Koveela Govindasami, Michelle Guy, Rosemary A. Wilkinson, Emma J. Sawyer, Angela Morgan, COGS-CRUK GWAS-ELLIPSE (Part of GAME-ON) Initiative, The UK Genetic Prostate Cancer Study Collaborators, The UK ProtecT Study Collaborators, The PRACTICAL Consortium, Douglas F. Easton, Ken Muir, Rosalind A. Eeles, Zsofia Kote-Jarai

**Affiliations:** 1 The Institute of Cancer Research, Sutton, Surrey, United Kingdom; 2 Department of Medical Epidemiology and Biostatistics, Karolinska Institute, Stockholm, Sweden; 3 Centre for Cancer Genetic Epidemiology, Department of Public Health and Primary Care, University of Cambridge, Strangeways Laboratory, Cambridge, United Kingdom; 4 Surgical Oncology (Uro-Oncology: S4), University of Cambridge, Addenbrooke's Hospital, Cambridge and Cancer Research UK Cambridge Research Institute, Li Ka Shing Centre, Cambridge, United Kingdom; 5 Nuffield Department of Surgical Sciences, University of Oxford, Oxford, and Faculty of Medical Science, University of Oxford, John Radcliffe Hospital, Oxford, United Kingdom; 6 School of Social and Community Medicine, University of Bristol, Bristol, United Kingdom; 7 Cancer Epidemiology Centre, The Cancer Council Victoria, Carlton, Victoria, Australia and Centre for Molecular, Environmental, Genetic and Analytic Epidemiology, The University of Melbourne, Melbourne, Victoria, Australia; 8 Department of Preventive Medicine, Keck School of Medicine, University of Southern California/Norris Comprehensive Cancer Center, Los Angeles, California, United States of America; 9 Program in Genetic Epidemiology and Statistical Genetics, Department of Epidemiology, Harvard School of Public Health, Boston, Massachusetts, United States of America; 10 Epidemiology Research Program, American Cancer Society, Atlanta, Georgia, United States of America; 11 Division of Cancer Epidemiology and Genetics, National Cancer Institute, NIH, Bethesda, Maryland, United States of America; 12 Nutritional Epidemiology Branch, National Cancer Institute, NIH, EPS-3044, Bethesda, Maryland, United States of America; 13 Division of Urologic Surgery, Washington University School of Medicine, St. Louis, Missouri, United States of America; 14 Department of Medic Biochemistry and Genetics, University of Turku, Turku and Institute of Biomedical Technology and BioMediTech, University of Tampere and FimLab Laboratories, Tampere, Finland; 15 Department of Clinical Biochemistry, Herlev Hospital, Copenhagen University Hospital, Herlev, Denmark; 16 Genomic Epidemiology Group, German Cancer Research Center (DKFZ), Heidelberg, Germany; 17 Division of Cancer Epidemiology, German Cancer Research Center (DKFZ), Heidelberg, Germany; 18 Department of Epidemiology & Biostatistics, School of Public Health, Imperial College London, London, United Kingdom; 19 Cancer Epidemiology Unit, Nuffield Department of Population Health, University of Oxford, Oxford, United Kingdom; 20 Cancer Prevention Institute of California, Fremont, California, United States of America, and Stanford University School of Medicine, Stanford, California, United States of America; 21 Division of Epidemiology, Department of Population Health, NYU Langone Medical Center, NYU Cancer Institute, New York, New York, United States of America; 22 Clinical Gerontology Unit, University of Cambridge, Cambridge, United Kingdom; 23 Department of Epidemiology, School of Public Health, University of Washington and Division of Public Health Sciences, Fred Hutchinson Cancer Research Center, Seattle, Washington, United States of America; 24 National Human Genome Research Institute, National Institutes of Health, Bethesda, Maryland, United States of America; 25 International Epidemiology Institute, Rockville, Maryland, and Division of Epidemiology, Department of Medicine, Vanderbilt Epidemiology Center, Vanderbilt-Ingram Cancer Center, Vanderbilt University School of Medicine, Nashville, Tennessee, United States of America; 26 Mayo Clinic, Rochester, Minnesota, United States of America; 27 Department of Urology, University Hospital Ulm and Institute of Human Genetics University Hospital Ulm, Ulm, Germany; 28 Division of Urologic Surgery, Brigham and Women's Hospital, Dana-Farber Cancer Institute, Boston, Massachusetts, United States of America; 29 International Hereditary Cancer Center, Department of Genetics and Pathology, Pomeranian Medical University, Szczecin, Poland; 30 Division of Genetic Epidemiology, Department of Medicine, University of Utah School of Medicine and George E. Wahlen Department of Veterans Affairs Medical Center, Salt Lake City, Utah, United States of America; 31 Division of Clinical Epidemiology and Aging Research, German Cancer Research Center (DKFZ), Heidelberg, Germany; 32 Department of Cancer Epidemiology, H. Lee Moffitt Cancer Center, Tampa, Florida, United States of America; 33 Molecular Medicine Center and Department of Medical Chemistry and Biochemistry, Medical University - Sofia, Sofia, Bulgaria; 34 Australian Prostate Cancer Research Centre-Qld, Institute of Health and Biomedical Innovation and School of Biomedical Science, Queensland University of Technology, Brisbane, Queensland, Australia; 35 Biomedical Sciences Institute (ICBAS), Porto University, Porto, and Department of Genetics, Portuguese Oncology Institute, Porto, Portugal; 36 Center for Cancer Genomics, Wake Forest University School of Medicine, Winston-Salem, North Carolina, United States of America; 37 Warwick Medical School, University of Warwick, Coventry, United Kingdom; Georgia Institute of Technology, United States of America

## Abstract

The *HOXB13* gene has been implicated in prostate cancer (PrCa) susceptibility. We performed a high resolution fine-mapping analysis to comprehensively evaluate the association between common genetic variation across the *HOXB* genetic locus at 17q21 and PrCa risk. This involved genotyping 700 SNPs using a custom Illumina iSelect array (iCOGS) followed by imputation of 3195 SNPs in 20,440 PrCa cases and 21,469 controls in The PRACTICAL consortium. We identified a cluster of highly correlated common variants situated within or closely upstream of *HOXB13* that were significantly associated with PrCa risk, described by rs117576373 (OR 1.30, *P* = 2.62×10^−14^). Additional genotyping, conditional regression and haplotype analyses indicated that the newly identified common variants tag a rare, partially correlated coding variant in the *HOXB13* gene (G84E, rs138213197), which has been identified recently as a moderate penetrance PrCa susceptibility allele. The potential for GWAS associations detected through common SNPs to be driven by rare causal variants with higher relative risks has long been proposed; however, to our knowledge this is the first experimental evidence for this phenomenon of synthetic association contributing to cancer susceptibility.

## Author Summary

Genome-wide association studies (GWAS) have identified numerous low penetrance disease susceptibility variants, yet few causal alleles have been unambiguously identified. The underlying causal variants are expected to be predominantly common; however synthetic associations with rare, higher penetrance variants have been hypothesised though not yet observed. Here, we report detection of a novel common, low penetrance prostate cancer association at the *HOXB* locus at ch17q and show that this signal can actually be attributed to a previously identified rare, moderate penetrance coding variant (G84E) in *HOXB13*. This study therefore provides the first experimental evidence for the existence of synthetic associations in cancer and shows that where GWAS signals arise through this phenomenon, risk predictions derived using the tag SNP would substantially underestimate the relative risk conferred and overestimate the number of carriers of the causal variant. Synthetic associations at GWAS signals could therefore account for a proportion of the missing heritability of complex diseases.

## Introduction

Prostate cancer (PrCa) is the most common cancer affecting men in developed countries, accounting for 25% of cancer diagnoses among males in the UK in 2010 (http://www.cancerresearchuk.org/cancer-info/cancerstats/types/prostate/incidence/). Whilst the majority of men will develop some form of prostate neoplasm during their lifetime, these are usually slow progressing and remain asymptomatic until their death; therefore only a proportion of prostate tumours require clinical intervention [1]. Currently, prostate specific antigen (PSA) is the only available biomarker for PrCa, however the specificity of this test for clinically significant disease is poor and its use for PrCa screening remains controversial; with little evidence of significant reduction in mortality and at the cost of substantial overdiagnosis and overtreatment of patients [2], [3]. Accordingly, much recent research has attempted to improve identification of individuals at greater risk of developing prostate tumours that require clinical intervention, to enable better application of treatment. Twin studies have suggested that PrCa has a substantial heritable component [4], whilst family history of PrCa among first degree relatives remains among the strongest known risk factors for the disease [5], [6]. As a result, many studies have looked for genetic variants that predispose towards the development of PrCa. Relatively few moderate penetrance risk variants for PrCa have been identified so far; however more than 70 common, low penetrance variants that individually modestly increase risk have been identified to date through GWAS studies [7], [8].

We recently reported 23 novel PrCa susceptibility SNPs identified through genotyping 20,440 PrCa cases and 21,469 controls from the PRACTICAL consortium on a custom Illumina iSelect array (iCOGS) [7]. These SNPs were all from loci that had shown some evidence for association with PrCa in our initial GWAS [9]; however the iCOGS array also contained a subset of SNPs that were included to examine possible associations with plausible PrCa susceptibility candidate genes. HOX genes are known to have crucial roles in development and previous evidence suggested their potential involvement in oncogenesis [10], including *HOXB13* specifically in PrCa [11], [12]. In addition, the *HOXB13* locus at chr17q21 was a region that had been previously implicated in PrCa susceptibility by linkage studies [13]–[15] and had been shown to undergo loss of heterozygosity in prostate tumours [16]–[18]. As a result, we targeted the *HOXB* cluster at chr17q21 to be densely genotyped on the iCOGS array. In addition, a closely situated ovarian cancer risk association around the *SKAP1* gene [19] had also been targeted for fine-mapping by the Ovarian Cancer Consortium (OCAC), providing additional SNPs covering this chromosomal region.

Whilst genotyping on the iCOGS array was being performed, Ewing *et al.* published evidence that a rare non-synonymous coding variant in *HOXB13* (G84E, rs138213197) was associated with hereditary prostate cancer (HPC) [20]. This risk variant has subsequently been confirmed to be a moderate penetrance susceptibility allele in a number of other studies and it was shown that the association was strongest with younger onset and familial PrCa [21]–[23]. Additional studies examining the geographical spread of the G84E variant have determined that it is observed almost exclusively in Caucasians and predominantly on the same haplotype background. This haplotype occurs more frequently in Nordic countries, most strikingly within the Finnish population, and suggests that rs138213197 is a founder mutation that arose relatively recently in Northern Europe [24], [25].

In this study, we show evidence that there is a cluster of novel common, low penetrance PrCa risk alleles in the *HOXB* region which appear to tag the rarer, moderate penetrance coding variant rs138213197. To our knowledge, this represents the first identified occurrence of a synthetic association in cancer.

## Results

After QC, 700 SNPs from the interval Chr17:46201311–47382559 (GRCh37/hg19) encompassing the *HOXB* locus were analysed on the iCOGS array in 20,440 cases and 21,469 controls of European ancestry from the PRACTICAL consortium. Two panel imputation was performed for the interval Chr17:46200000–47400000 using a 1000 Genomes Phase 1 integrated variant set and Illumina OMNI2.5 BeadChip data for 677 PrCa cases from the UKGPCS study. This generated imputed data for 3195 SNPs with MAF≥0.01 within this region in the iCOGS sample set. These thresholds do not retain imputation information for the previously reported coding variant (G84E, rs138213197) due to its low MAF. As this SNP is a reported PrCa susceptibility variant and was in close proximity to a cluster of variants showing association with PrCa risk in our imputed data, additional genotyping was carried out by Taqman and Sequenom assays for this SNP in 5500 cases and 4923 controls from the UK and Sweden. We subsequently attempted to impute the rs138213197 variant to the entire iCOGS sample set using this additional panel, however the imputation quality remained inadequate and therefore analyses involving this variant were performed on the directly genotyped subset of samples only.

**Figure 1:**
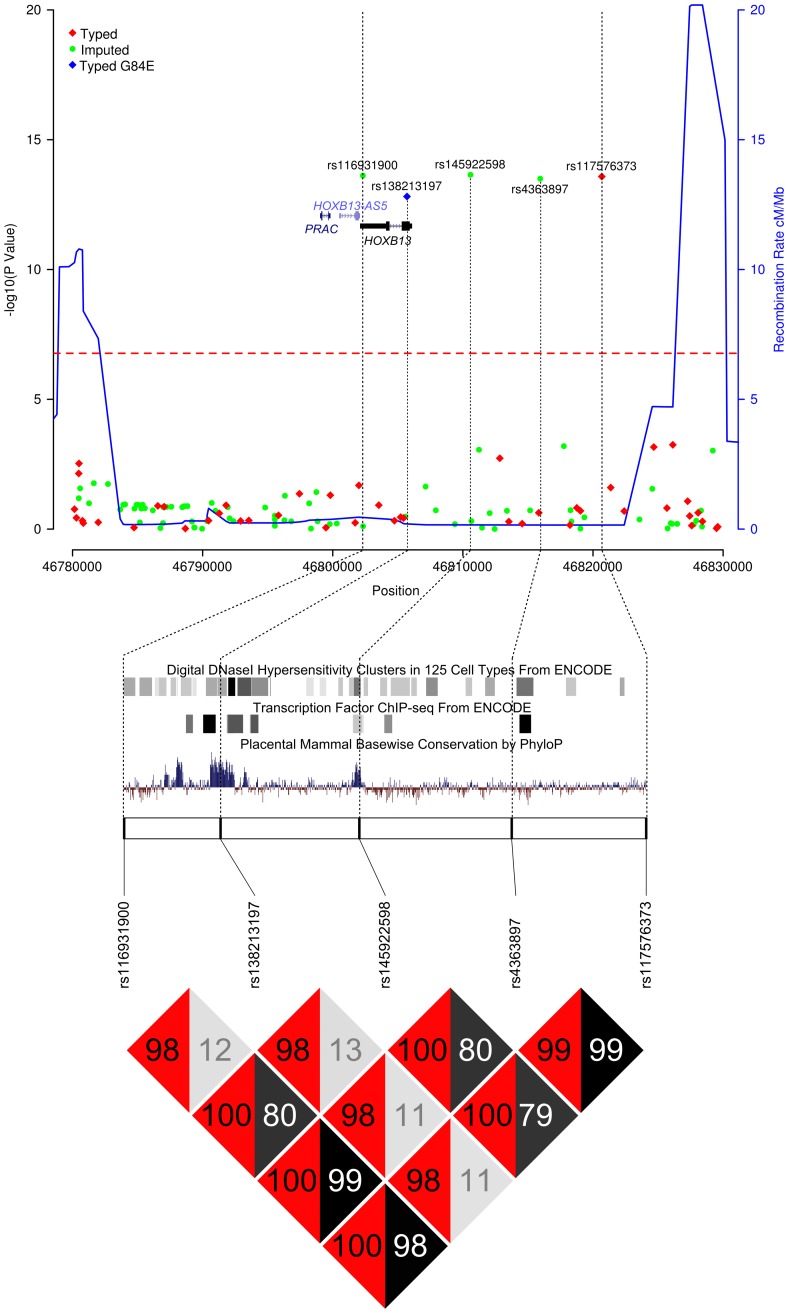
Results of the *HOXB* locus fine-mapping analysis. Upper Panel – Regional association plot of SNPs at the *HOXB13* locus. Association data from the iCOGS dataset of 20,440 PrCa cases and 21,469 controls are shown with genotyped SNPs in red and imputed SNPs in green. The Bonferroni-adjusted level of significance is denoted by the red line. The G84E variant rs138213197 was genotyped in a smaller subset of 5500 PrCa cases and 4923 controls and is marked by the blue rectangle. Also indicated are the position of genes within this interval and the location of neighbouring recombination hotspots. Middle Panel – Intersection between the 5 SNPs significantly associated with PrCa and putative functional elements identified by the ENCODE project or regions of mammalian sequence conservation by PhyloP. Lower Panel – Pairwise Linkage Disequilibrium values for the 5 SNPs significantly associated with PrCa. *r^2^* values are shown in grey and D′ in red.

Following imputation, four SNPs in close proximity to one another and situated within or closely upstream of the *HOXB13* gene remained significantly associated with PrCa at *P*<10^−6^ (Table 1); of which one, rs117576373, had been genotyped on the iCOGS array. This cluster of variants are highly correlated (*r^2^*≥0.79) and envelop the published missense coding variant rs138213197 (G84E) (Figure 1). In the subset of samples that had been genotyped for rs138213197, the correlation between rs138213197 and the cluster of variants represented by rs117576373 initially appears very modest (*r^2^*≤0.13, Figure 1), implying that the newly identified cluster of variants represented a novel association signal. However, the MAFs of these variants are substantially different (2.6–4.3% vs. 0.4% in our control set), with the rare allele of rs138213197 (T) almost exclusively co-inherited with the minor allele of rs117576373 (T) (D′ 0.98). The nature of the correlation between these variants could therefore be consistent with rs117576373 representing an additional novel, common, lower penetrance association signal at the *HOXB13* region that is almost invariably present alongside the moderate penetrance rs138213197 variant but is also found by itself in a greater number of individuals. Alternatively, in spite of the low *r^2^* between the two variants, the novel association signal could in fact be tagging the rare G84E variant, thereby resulting in detection of a synthetic association signal at a common variant that is in fact mediated by a much rarer causal variant.

**Table 1:** PrCa association information for SNPs significant at *P*<10^−6^ in the iCOGS imputed data.

SNP	Pos (Chr17)	MAF#	Method	$Uni *P*	$Uni OR	*Uni *P*	*Uni OR	*Multi *P*	*Multi OR
rs116931900	46802314	0.038	Imputed	2.42×10^−14^	1.31 (1.22–1.40)	4.22×10^−5^	1.31 (1.15–1.50)	0.72	1.17 (0.50–2.73)
rs138213197	46805705	0.004	Typed	-	-	1.54×10^−13^	3.72 (2.62–5.27)	4.2×10^−12^	3.88 (2.64–5.70)
rs145922598	46810586	0.026	Imputed	2.25×10^−14^	1.38 (1.27–1.50)	4.98×10^−4^	1.31 (1.13–1.53)	0.025	1.50 (1.05–2.13)
rs4363897	46815947	0.039	Imputed	3.17×10^−14^	1.32 (1.23–1.42)	4.23×10^−5^	1.32 (1.16–1.52)	0.53	1.47 (0.45–4.86)
rs117576373	46820676	0.043	Typed	2.62×10^−14^	1.30 (1.22–1.40)	2.28×10^−5^	1.32 (1.16–1.52)	0.28	1.74 (0.64–4.71)

**Uni** denotes data from univariate analyses and **Multi** the results after conditional regression analysis. rs138213197 could not be accurately imputed into the iCOGS sample set and was analysed in directly genotyped samples only.

# Minor Allele Frequency in our control sample set.

$ Analyses were performed on the full iCOGS data set of 20,440 cases and 21,469 controls.

* Analyses were performed on the subset of 5500 cases and 4923 controls genotyped for both rs138213197 and rs117576373.

To elucidate which of these scenarios explain the PrCa risk association at this locus we first performed a conditional regression analysis for rs138213197 and the cluster of newly identified variants, using the subset of cases that had been genotyped for both rs138213197 and rs117576373. rs138213197 remained highly significant in this analysis (*P* = 4.2×10^−12^, Table 1) and with an effect size substantially greater than that observed through any of the common variants and broadly similar to that previously reported in the literature (OR = 3.88, 95%C.I. 2.64–5.70). This suggests that the association with PrCa risk arises predominantly through this rare coding variant. In addition, one of the more common SNPs, rs145922598, also exhibited some evidence for association (*P* = 0.025). This SNP is somewhat less frequent than the other three SNPs in the newly identified cluster, however is still highly correlated with these (*r^2^*∼0.8, D′∼1) and is located in a region of high conservation and functional context (Figure 1); therefore could potentially represent a novel low penetrance association signal. To further examine whether these variants represent the same or separate association signals, in the subset of samples in which the coding variant had been directly genotyped, we conducted haplotype analyses between rs138213197 and rs117576373 (both directly genotyped) and additionally between rs138213197 and rs145922598 (imputed). This provides further confirmation that rs138213197 is most likely responsible for PrCa risk alone; since the moderately frequent rs138213197 (C, major allele)–rs117576373 (T, minor allele) and rs138213197 (C, major allele)–rs145922598 (T, minor allele) haplotypes showed no evidence for association with disease risk, and a positive association with risk was only observed in haplotypes where the rs138213197 (T, minor allele) risk allele was present (Tables 2 & 3).

**Table 2:** Haplotype analysis for rs117576373 and rs138213197 in the subset of 5500 PrCa cases and 4923 controls from the UK and Sweden for which both had been directly genotyped.

rs117576373	rs138213197	Haplotype	Case Freq	Control Freq	P	OR	$Empirical *P*	$Corrected *P*
T	T	Minor | Minor	0.015	0.004	1.85×10^−17^	4.16 (2.91–5.94)	9.99×10^−5^	1.00×10^−4^
T	C	Minor | Major	0.038	0.037	0.74	1.02 (0.89–1.20)	0.74	1.00
C	T	Major | Minor	0	0	-	-	-	-
C	C	Major | Major	0.947	0.959	3.6×10^−5^	0.76 (0.67–0.87)	9.99×10^−5^	1.00×10^−4^

$ **Empirical** ***P*** values were generated after 1000 permutations testing of case control status. **Corrected** ***P*** values were subsequently generated from these by adjusting for multiple testing.

**Table 3:** Haplotype analysis for rs145922598 and rs138213197 in the subset of 5500 PrCa cases and 4923 controls from the UK and Sweden genotyped for rs138213197.

rs145922598	rs138213197	Haplotype	Case Freq	Control Freq	P	OR	$Empirical *P*	$Corrected *P*
T	T	Minor | Minor	0.015	0.004	7.47×10^−15^	3.76 (2.63–5.38)	1.00×10^−4^	1.00×10^−4^
T	C	Minor | Major	0.021	0.022	0.77	0.98 (0.87–1.11)	0.78	1.00
C	T	Major | Minor	0	0	-	-	-	-
C	C	Major | Major	0.964	0.974	0.01	0.86 (0.77–0.97)	0.01	0.03

rs145922598 genotype information was extracted from our imputed dataset.

$ **Empirical** ***P*** values were generated after 1000 permutations testing of case control status. **Corrected** ***P*** values were subsequently generated from these by adjusting for multiple testing.

In addition to this novel association signal, we also confirmed a previously reported association within our imputation interval described by rs11650494 in Caucasians [7] and rs7210100 in African Americans [26]. This signal is situated >500 kb downstream of the novel variant cluster (Supplementary Figure S1), around the *ZNF652* gene. We observed no significant linkage disequilibrium between these two clusters of variants (*r^2^* = 0, D′≈0.02) and our conditional analysis confirmed that they represent separate associations with PrCa.

## Discussion

In this study, we identified a novel common PrCa association signal at the *HOXB13* locus. Further investigation revealed that this signal is most likely to arise due to correlation with the previously reported rare, moderate penetrance coding variant rs138213197. Despite the fact that in this instance the rare, putative causal variant was discovered prior to that of the more common tag SNP, this PrCa susceptibility locus still serves as a useful illustration of the potential range of causal variation underpinning GWAS association signals, as well as the potential pitfalls of attempting to elucidate candidate causal variants.

rs138213197 was discovered through re-sequencing of a PrCa linkage hit at chr17q21 in hereditary prostate cancer (HPC) families; although as we show here, it could instead have been indirectly detected through the modestly correlated variant rs117576373 in a suitably powered GWAS study with sufficient marker density such as the iCOGS study. Due to the large difference in allele frequency between the tag and causal SNPs, and the inability to accurately impute the rare variant even with *a priori* knowledge of its existence, discovering this susceptibility locus by this route would therefore disguise the contribution of the rare variant and artificially diminish the observed relative risk; which would consequently have implicated the likely causal variant(s) to be relatively common, low penetrance and tightly correlated with the typed SNP rs117576373. However, while we cannot completely exclude that common variation may contribute to PrCa risk at this locus as data from the ENCODE project suggests some degree of potential functionality for the variants we have identified here, (in particular rs145922598, which remained marginally significant in the conditional regression, is highly conserved, overlaps a DNaseI hypersensitivity site in several cell lines including LNCaP and transcription factor binding sites for FOXA1 and FOXA2 transcription factors (Table 1, Figure 1)); the much stronger evidence for significance for the rare coding variant coupled with the results of our haplotype analyses appear to indicate that this SNP is solely responsible for the detected association signal. As such, this appears to be an example of a rare variant with a sufficiently large effect size to create a synthetic association signal detected through partially correlated yet significantly more common variants. It is also worth noting that had the rs138213197 variant not been previously identified as a PrCa susceptibility variant, it would have been unlikely to have been discovered during this imputation based fine-mapping approach since the MAF of this SNP is below conventional QC thresholds for imputation; indeed, it remains poorly imputed even using a two panel method in which a subset of samples had been directly genotyped for this variant. The potential consequence of this inability to accurately impute low frequency variants is that the search for candidate causal variants for functional follow-up would be inevitably skewed towards common variation. Furthermore, as we have observed here, where rare causal variants underpin an association signal, risk effect size estimates may consequently be significantly underestimated and the assumed proportion of carriers of the causal variant inflated. Our observations therefore provide support for the suggestion that identifying the actual causal variants behind GWAS associations could account for a proportion of the missing heritability in common diseases and that re-sequencing of GWAS loci in large numbers of cases and controls would be important for the discovery of the full spectrum of correlated variation.

The nature of the underlying genetic architecture behind GWAS signals has been the subject of much debate. Whilst few causal alleles have been unambiguously categorised, several authors have presented evidence that suggests common variants are likely to comprise the vast majority of these [27]–[29]. Conversely, computational analyses have demonstrated that rare causal variation has the potential capability to give rise to the GWAS signals detected through more common variants [30]–[32]. For PrCa, fine-mapping and functional evidence at a handful of risk loci appears to implicate common SNPs as the most likely candidate causal variants at these regions. For example, at the *MSMB* region at chr10q11, the common GWAS tag SNP situated in the *MSMB* promoter remained the most plausible candidate causal variant after fine-mapping by sequencing [33], with functional studies also demonstrating that the risk allele disrupts a transcription factor binding site, resulting in decreased expression of *MSMB* [34]. We have also performed fine-mapping studies by imputation for the *KLK* region at chr19q13 and *TERT* locus at chr5p15. We identified a common missense coding SNP in the *KLK3* gene that was more strongly associated with PrCa than the original tag SNP and represents a candidate causal variant for this association [35], whilst four independently associated clusters of common variants were described at the *TERT* locus [36]; however these studies were not powered to detect any contribution by rare variants and despite refining the original associations, have not unambiguously established the causal allele(s) at these regions. This study therefore provides the first direct evidence of which we are aware for a substantial contribution of rare variation to an association signal for PrCa. This suggests that it is entirely plausible that both mechanisms may indeed give rise to GWAS associations, and need not necessarily be mutually exclusive. Logically however, the higher the MAF of the tag SNP at a susceptibility locus the greater the likelihood that the associated causal variant(s) will be common, whilst synthetic associations would become increasingly plausible at lower index SNP MAFs (in this study 2.6–4.3% MAF). Furthermore, whilst this study does provide experimental evidence for the existence of synthetic associations, no inference can be made as to how frequently they might account for the causal variant behind the numerous disease associations that GWAS have discovered. However, by capitalising upon the differences in genetic architecture between different ethnic populations in addition to the steadily increasing quantities of sequencing data that are becoming available to the research community, this may become more clear and help to guide future fine-mapping studies. In particular, as the causal alleles behind synthetic associations are rare, these associations are more likely to be limited to specific ethnic groups and therefore the absence of a multi-ethnic signal for a tag SNP of modest frequency could indicate a greater likelihood that re-sequencing the locus would identify rare causal variation.

In summary, this study provides evidence for several widely discussed concepts regarding the nature of causal variation at GWAS hits and their contribution to the heritability of common diseases. Firstly, we have shown that low frequency, moderate penetrance susceptibility variants can be detected via common tag SNPs in GWAS studies when there is little recombination between these variants. Secondly, that imputation based fine mapping alone is likely to implicate candidate causal variants as common, some of which may have plausible biological function; therefore sufficiently powered re-sequencing of loci is ultimately desirable to assess and possibly exclude the contribution of rare variants. Finally, that for GWAS associations where the tag SNP is correlated with a rare causal variant, the relative risk estimates derived from the tag SNP are likely to be considerably underestimated, which could in turn account for a proportion of the missing heritability of common diseases.

## Methods

### Samples

Samples for the iCOGS study were drawn from 25 studies participating in the PRACTICAL Consortium [7]. The majority of studies were population-based or hospital-based case-control studies, or nested case-control studies. All studies have the relevant IRB approval in each country in accordance with the principles embodied in the Declaration of Helsinki. After exclusion of samples that failed quality control (QC) in the iCOGS study or showed substantial non-European ancestry, genotype data for 20,440 PrCa cases and 21,469 matched controls were available.

To improve imputation performance, Illumina OMNI2.5 SNP array data were available for 677 UK PrCa cases from the UKGPCS study (www.icr.ac.uk/ukgpcs); 262 of these cases were also genotyped on the iCOGS array. The rare coding variant rs138213197 was also genotyped in 2476 cases and 2198 controls from the UK (UKGPCS study), and 3024 cases and 2725 controls from Sweden (CAPS and STHM1 study).

### Genotyping

Detailed information relating to the custom iCOGS Illumina Infinium array can be found in Eeles *et al.*, 2013 [7]. With respect to the *HOXB* locus, 747 SNPs spanning the interval chr17:46201311–47382559 were genotyped on the iCOGS array, submitted by a combination of the PRACTICAL and OCAC consortia (Supplementary Figure S1).

To boost imputation performance, additional genotyping of 677 PrCa cases from the UK was conducted using the Illumina (San Diego, CA, USA) OMNI2.5 BeadChip according to the manufacturer's instructions. Further genotyping of the rs138213197 variant was carried out by Taqman assay (Applied Biosystems Inc., Foster City, CA, USA) for 2476 cases and 2198 controls from the UK and by MassARRAY iPLEX (Sequenom Inc., San Diego, CA, USA) for 3024 cases and 2725 controls from Sweden.

### Imputation

Imputation was performed on 20,440 case and 21,469 control samples across the 700 iCOGS SNPs from the *HOXB13* interval which passed pre-imputation QC metrics [37] (Supplementary Figure S2). IMPUTE v2.3.0 [38], [39] was used to impute the interval Chr17:46200000–47400000 (GRCh37/hg19). Two panel imputation [40] was performed using OMNI2.5 BeadChip data for 677 PrCa cases from the UKGPCS study (Panel 1) and a 1000 Genomes Phase 1 integrated variant set “version 3” (SNPs and in/dels) from 5^th^ March 2012 (Panel 0) (http://mathgen.stats.ox.ac.uk/impute/data_download_1000G_phase1_integrated.html). Imputation concordance was examined using “leave one out” internal concordance check. For single panel imputation (Panel 0 only) concordance was 96.5% at SNPs *r^2^*≥0.5 and 98.0% at *r^2^*≥0.9, which rose to 98.9% at *r^2^*≥0.5 and 99.8% at *r^2^*≥0.9 respectively for two panel (Panel 0+1) imputation. SNPs with info <0.5, MAF<0.01 were excluded during QC filtering.

### Statistical Analysis

Association tests were performed on genotypes in the MaCH dosage format (0–2) converted from the IMPUTE genotype posterior probabilities using GenABEL [41]. Associations between each SNP and PrCa risk were analysed using a per-allele trend test, adjusted for study and six principal components derived from analysis of the whole iCOGS dataset [7]. Odds ratios (OR) and 95% confidence limits were estimated using unconditional logistic regression. Tests of homogeneity of the ORs across strata were assessed using a likelihood ratio test. SNPs significant at *P*<10^−6^ were considered for further analysis. The independence of these associations was assessed by performing a conditional logistic regression analysis. For further assessment of the relationship between rs138213197 and rs117576373, haplotype analyses were performed with Plink 1.07 (http://pngu.mgh.harvard.edu/purcell/plink/) on the subset of samples where both SNPs had been directly genotyped. Haplotypes were first imputed using the Expectation-Maximisation algorithm in order to then perform a case-control association, using 10,000 permutations of the phenotype labels [42].

## Supporting Information

Figure S1 Distribution of genotyped SNPs at the *HOXB* locus on chromosome 17 on the iCOGS array. The position of the *HOXB13* gene is indicated by the blue rectangle. Two clusters of variants significantly associated with PrCa were identified. The cluster marked in green represents a previously reported low penetrance association signal described by the typed SNP rs11650494 (Eeles *et al.*, 2013, Nature Genetics) and are not discussed further within the scope of this manuscript. The cluster of four SNPs marked in red represented a novel association signal. There is no significant linkage disequilibrium between these clusters of variants (*r^2^* = 0, D′≈0.02).
(PNG)

Figure S2 Flowchart detailing the two panel imputation process used to impute the *HOXB* locus at chromosome 17 in PrCa cases and controls from the PRACTICAL consortium. The 1000 Genomes Project dataset used for imputation was a March 2012 “version 3” of the Phase 1 integrated data.
(PNG)

Supplementary Information S1 List of members of consortia that have contributed to this work.
(DOCX)

Supplementary Information S2 Details of additional funding, abbreviations and URLs.
(DOCX)
