# Supplementary figures and images for: Fine-Mapping the HOXB Region Detects Common Variants Tagging a Rare Coding Allele: Evidence for Synthetic Association in Prostate Cancer
Source: PLoS Genet. 2014 Feb 13;10(2):e1004129. doi: 10.1371/journal.pgen.1004129 (PMC3923678; doi:10.1371/journal.pgen.1004129)

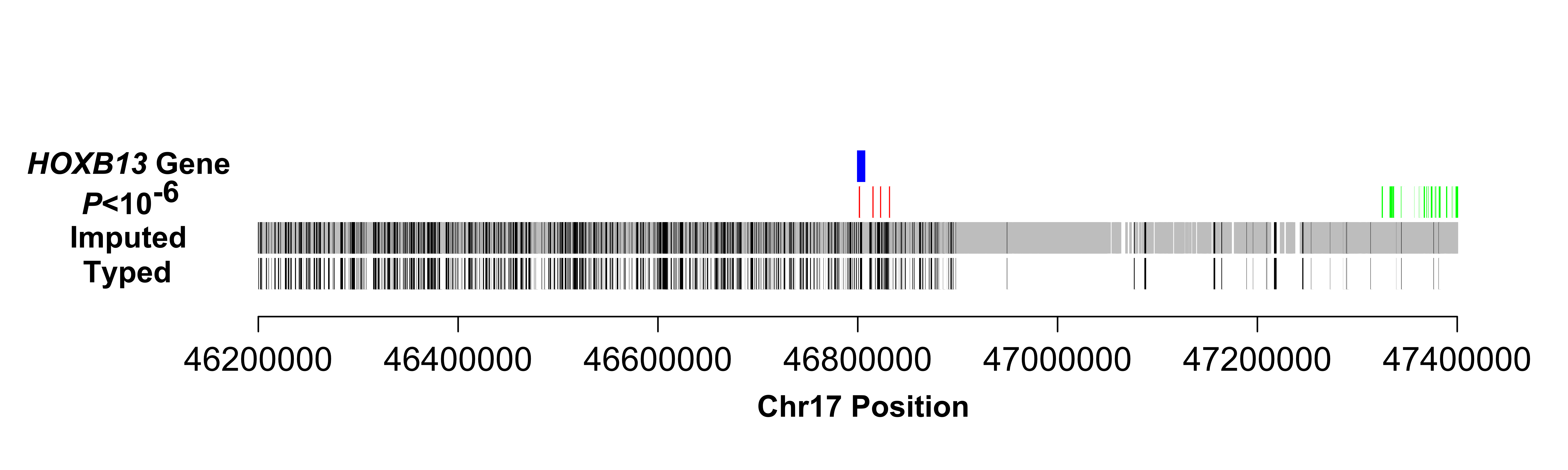

Supplement: Figure S1 — Distribution of genotyped SNPs at the HOXB locus on chromosome 17 on the iCOGS array. The position of the HOXB13 gene is indicated by the blue rectangle. Two clusters of variants significantly associated with PrCa were identified. The cluster marked in green represents a previously reported low penetrance association signal described by the typed SNP rs11650494 (Eeles et al., 2013, Nature Genetics) and are not discussed further within the scope of this manuscript. The cluster of four SNPs marked in red represented a novel association signal. There is no significant linkage disequilibrium between these clusters of variants (r2 = 0, D′≈0.02). (PNG) [file pgen.1004129.s001.png]

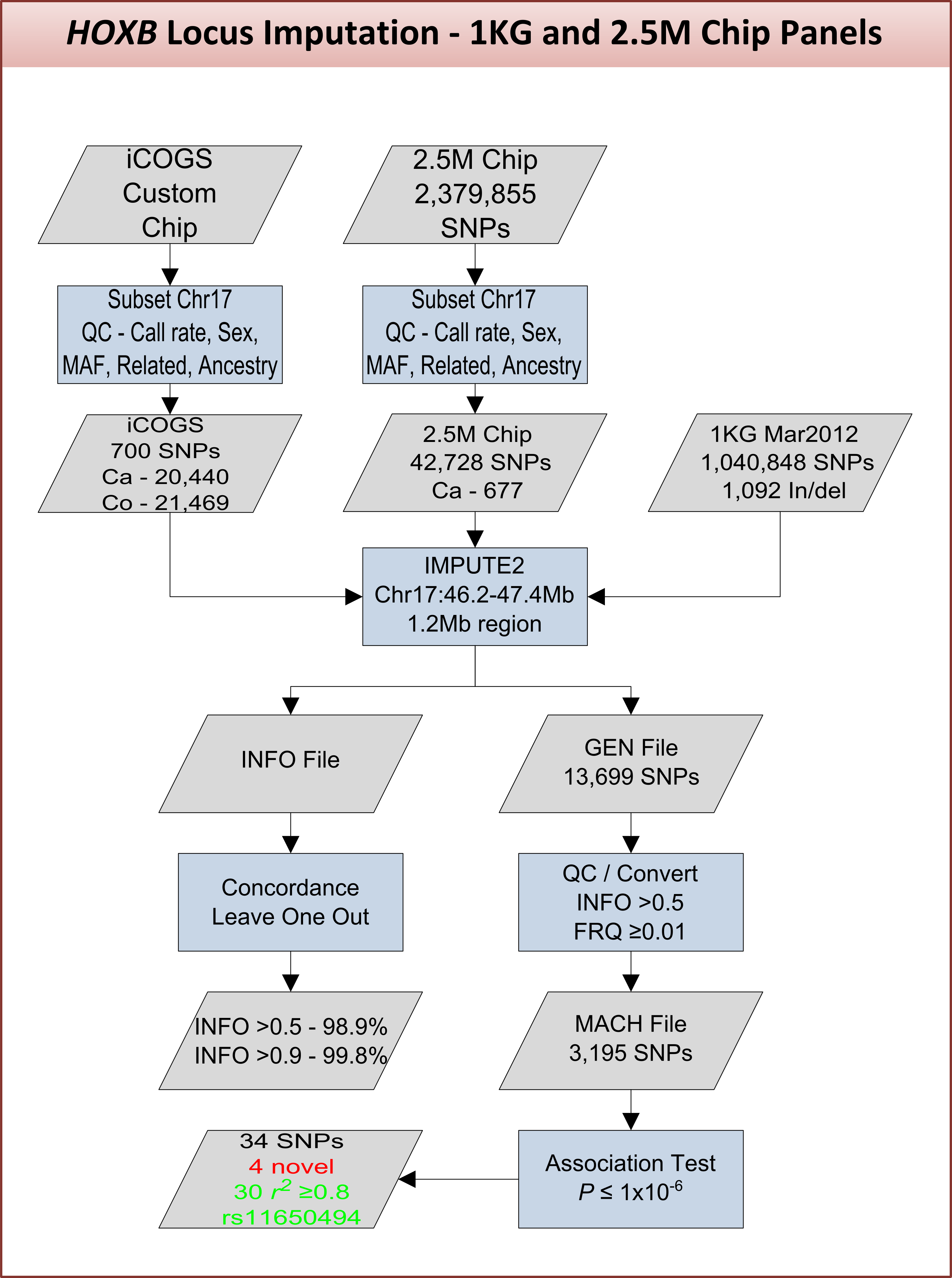

Supplement: Figure S2 — Flowchart detailing the two panel imputation process used to impute the HOXB locus at chromosome 17 in PrCa cases and controls from the PRACTICAL consortium. The 1000 Genomes Project dataset used for imputation was a March 2012 “version 3” of the Phase 1 integrated data. (PNG) [file pgen.1004129.s002.png]
