# Supplementary material for: Fine-Mapping the HOXB Region Detects Common Variants Tagging a Rare Coding Allele: Evidence for Synthetic Association in Prostate Cancer
Source: PLoS Genet. 2014 Feb 13;10(2):e1004129. doi: 10.1371/journal.pgen.1004129 (PMC3923678; doi:10.1371/journal.pgen.1004129)
Supplement: Supplementary Information S1 — List of members of consortia that have contributed to this work. (DOCX) [file pgen.1004129.s003.docx]

**Supplementary Information S1**

**List of members of consortia that have contributed to this work.**

**UKGPCS, ProtecT and PRACTICAL consortium member lists**

**The UK Genetic Prostate Cancer Study Collaborators**

Mr Z Abbasi, Mr M Akhlil Abdul-Hamid, Mr Paul D Abel, Professor Paul H Abrams, Dr Fawzi A Adab, Mr Andrew Adamson, Mr A Adeyoju, Mr Naveed Afzal, Mr Ernest K N Ahiaku, Mr Munir Ahmed, Mr Mohammed L Al Sudani, Dr Christopher Alcock, Dr Zulfiqar Ali, Mr David J Almond, Dr Roberto Alonzi, Dr Amir S M Al-Samarraie, Dr Al-Samerraie, Mr Waleed Al-Singary, Mr Al-Sudani, Mr John Anderson, Mr Steven Andrews, Mr Henry Andrews, Mr Iqbal Anjum, Mr Ken Anson, Dr Nicola A Anyamene, Mr Ike Apakama, Dr F Aparcia, Mr J A A Archbold, Dr D Ash, Dr Richard F U Ashford, Dr A Azzabi, Mr David Badenoch, Dr Amit Bahl, Mr M J Bailey, Mrs Karen Bailey, Mr Andrew J Ball, Mr G Banerjee, Dr N Barber, Dr Jim Barber, Dr Baria, Mr Douglas G Barnes, Mr J Bashir, Mr Pradip Basu, Mr Christopher A Bates, Dr N A Bax, Mr D Baxter-Smith, Mr Amar Bdesha, Mr Christopher J M Beacock, Professor Ronald P Beaney, Mr Ralph Beard, Mr John D Beatty, Mr Rupert Beck, Ms Gail Beese, Dr Sharon Beesley, Mr C Richard W Bell, Mr James Bellringer, Dr Richard Benson, Dr Beresford, Mr Christopher R A Bevis, Dr Rajanee Bhana, Mr S Bhanot, Dr A Bhatnagar, Mr R I Bhatt, Mr Brian Birch, Dr Alison Birtle, Mr M Bishop, Mr C Shekhar Biyani, Mr A R E Blacklock, Miss Rosemary Blades, Dr Peter Bliss, Dr David J Bloomfield, Miss S Boddy, Professor C M Booth, Mr Pradeep Bose, Dr Michael C Bott, Dr David Bottomley, Mr Nigel R Boucher, Dr J Bowen, Dr Mark Bower, Mr W G Bowsher, Mr P J R Boyd, Mr F James Bramble, Mr Simon F Brewster, Mr Tim Briggs, Dr Cathryn Brock, Dr Sue Brock , Mr Stephen Bromage, Mr Richard Brough, Dr Richard Brown, Mr Stephen Brown, Mr Richard Brown, Mr Tony J Browning, Mr N Bryan, Mr Neil A Burgess, Mr Nicholas Burns-Cox, Mr Paul C Butterworth, Mr D Cahill, Mr P S Callaghan, Mr John Calleary, Dr M Calleja, Dr Frances Calman, Dr Philip Camilleri, Mr Alister Campbell, Miss Andrea Cannon, Dr Dawn M Carnell, Mr T W Carr, Mr Simon Carter, Mr Charles J M Carter, Dr Adam C Carter, Dr Bruce M Castle, Mr David Chadwick, Mr Rohit Chahal, Dr P Chakraborti, Mr Chappell, Mr C Charig, Dr Anula D Chetiyawardana, Mr Christopher Chilton, Mr F I Chinegwundoh, Dr Irene Chong, Dr Ananya Choudhury, Mr Wai-Man Chow, Mr Timothy J Christmas, Dr Mark J Churn, Mr Noel W Clarke, Mr Jorge Clavijo-Eisele, Dr M Coe, Mr N P Cohen, Mr C Coker, Dr Trevor Cole, Dr David J Cole, Mr O Cole, Mr Gerald Collins, Dr Matthew Collinson, Mr I Conn, Dr C Connell, Dr Audrey Cook, Mr Peter Cooke, Mr Graeme Cooksey, Mr L Coombs, Mr Robert F Copland, Mr Andrew J Cornaby, Mr P A Cornford, Mr Corolis, Mr John Corr, Mr C B Costello, Mrs N Coull, Dr Richard Cowan, Mr Robert Cox, Dr C Coyle, Mr Jeremy Crew, Mr John C Crisp, Dr W Cross, Mr W Cross, Dr Dorthe Cruger, Mr Malcolm Crundwell, Mr Cummings, Mr Nazeer Dahar, Dr Francis N Daniel, Mr J Darrad, Mr Pallon Daruwala, Mr Gautam Das, Mr Shibendra Datta, Dr S Davidson, Dr Joseph Davies, Mr Owen W Davison, Mr Guy Dawkins, Mr Chris Dawson, Mr Alan R De Bolla, Professor David Dearnaley, Mr Ken M Desai, Dr George P Deutsch, Mr John Dick, Mr Andrew J Dickinson, Dr Jeanette Dickson, Mr Michael Dinneen, Dr Sanjay Dixit, Dr H Jane Dobbs, Mr A Doble, Dr David Dodds, Mr Alan Doherty, Mr P Donaldson, Dr M Dooldeniya, Dr S Fiona Douglas, Mr Drake, Dr Gill M Duchesne, Mr Peter Duffy, Mr Michael Dunn, Mr W D Dunsmuir, Dr Sajid K Durrani, Mr Alan C Eaton, Professor Diane Eccles, Mr B Eddy, Mr C D Eden, Mr J Edwards, Mr Jeremy Elkabir, Dr P Tony Elliott, Mr B W Ellis, Dr R Ellis, Dr A El-Modir, Mr Andrew W S Elves, Dr Christine Elwell, Mr Mark Emberton, Dr Louise Emmerson, Mr Roland C D England, Mr R D Errington, Professor D Gareth Evans, Dr Alison Falconer, Mr Derek Fawcett, Dr C Featherston, Dr Carolyn J Featherstone, Mr Jeremy Feggetter, Dr C Ferguson, Dr D Fermont, Mr Michael Ferro, Mr Matthew Fletcher, Dr A Folkes, Mr Trevor F Ford, Mr Paul W Foster, Dr Kevin N Franks, Dr Olivera Frim, Dr Joanna Gale, Mr Christopher Gallegos, Mr James S Gelister, Dr Ghana, Dr Stephanie Gibbs, Mr Hugh Gilbert, Mr David Gillatt, Dr John Glaholm, Mr Jonathan M Glass, Mr James Glenister, Dr Thomas D Goode, Ms E M Gordon, Mr Richard L Gower, Dr John Graham, Mr Damian Green, Mr Jonathan Greenland, Dr Robert Grieve, Mr Thomas R L Griffiths, Mr Sandy Gujral, Dr Nishi Gupta, Mr Riza Murat Gurun, Mr Peter J Guy, Mr Neil Haldar, Mr N Halder, Professor F C Hamdy, Dr C Hamilton, Mr John Hammonds, Mr S J Hampson, Mr Damien C Hanbury, Dr P D John Hardman, Dr Stephen J Harland, Mr John M Harney, Dr Peter Harper, Dr Sarah Harris, Mr D Harris, Mr G S M Harrison, Mr D R Harriss, Mr N Harvey-Hills, Mr Simon Hawkyard, Dr Catherine M Heath, Mr Michael Hehir, Mr Giles O Hellawell, Mr David Hendry, Mr Mike Henley, Dr Ann Henry, Dr John Hetherington, Dr Tamas Hickish, Mr James A Hicks, Dr Serena Hilman, Mr Richard Hindley, Mr John R Hindmarsh, Mr John Hines, Dr M Hingorani, Mr Edwin T S Ho, Professor Shirley Hodgson, Dr U Hoffman, Mr David Holden, Dr A Hollingdale, Mr Graham W Hollins, Mr Simon A V Holmes, Dr Gail Horan, Professor Alan Horwich, Professor Peter Hoskin, Mr Graham P Howell, Mr D Hrouda, Dr Robert Huddart, Ms Liz Hudson, Dr Rob Hughes, Mr Michael Hughes, Mr Owen Hughes, Dr Caroline Humber, Mr John W Iacovou, Dr A Ibrahim, Mr John A Inglis, Mr Stuart Irving, Mr C Irwin, Dr Louise Izatt, Mr Victor Izegbu, Mr Basharat Jameel, Mr Michael J James, Professor N James, Mr R Lester James, Mr Pradip Javle, Dr P Jenkins, Dr Sameer Jhavar, Dr Gareth Jones, Mr Chris R Jones, Dr David A Jones, Mr J Joseph, Dr Shelagh Joss, Mr Amir Kaisary, Dr Alexandre L Kaliski, Dr G Kapur, Mr O Karim, Dr Stephen J Karp, Mr F X Keeley, Mr Anand R Kelkar, Mr J P Kelleher, Mr John Kelly, Dr Sue Kenwrick, Mr F Khan, Dr Vincent Khoo, Ms Rachel M Kimber, Mr R Kinder, Professor Roger S Kirby, Professor David Kirk, Dr Peter Kirkbride, Mr Magdi M Kirollos, Mr Roger Kockelbergh, Mr Philip C W C Koenig, Mr Gordon G Kooiman, Dr O Koreich, Mr Anthony Koupparis, Mr Mohamed Kourah, Dr Sigurd Kraus, Ms Magda L Kujawa, Mr Ravi Kulkarni, Mr M Kumar, Dr Ian H Kunkler, Professor H Kynaston, Dr Katherine L Lachlan, Dr Robert Laing, Dr Fiona Lalloo, Mr M Lancashire, Mr Stephen E M Langley, Mr Marc Laniado, Mr T R Larner, Mr Maurice W Lau, Mr W T Lawrence, Miss Anne Lawson, Mr Pieter J Le Roux, Professor Mary Leader, Mr J O Lee, Ms L Lee, Ms A Lee, Dr R John Lemburger, Dr Priscilla Leone, Dr Jason Lester, Mr Hing Leung, Mr J Lewis, Mr D Christopher Lewis, Mr Thomas Liston, Dr Jacqueline Livsey, Mr S Lloyd, Dr Imogen Locke, Mr Richard Lodge, Dr John Logue, Mr Mark Longmuir, Mr Malcolm G Lucas, Mr C J Luscombe, Dr Anna Lydon, Mr Michael Lynch, Mr Naing N K Lynn, Mr James P A MacDermott, Mr Ruaraidh P Macdonagh, Mr Macdonald, Mr Sanjeev Madaan, Dr Kudingila R Madhava, Dr Joseph Maguire, Professor E R Maher, Dr Rana Mahmood, Dr Graeme H M Mair, Mr Peter R Malone, Dr Stephen A Mangar, Mr Mark Mantle, Mr I Mark, Mr Robert Mason, Professor M D Mason, Mr Matanhelia, Mr Shyam Matenhelia, Mr Philip N Matthews, Dr J McAleese, Ms Donna McBride, Mr Jonathan McFarlane, Mr McGrath, Mr Craig McIlhenny, Mr Paul McInerney, Mr Gregor McIntosh, Dr F McKinna, Dr Duncan McLaren, Miss Esther McLarty, Dr Rhona McMenemin, Mr Alan McNeill, Mr T A McNicholas, Mr Robert N Meddings, Mr A David Mee, Dr Lucinda Melcher, Mr Memon, Mr Pravin Menzes, Mr Marek Miller, Mr Robert Mills, Mr S Mitchell, Dr Natasha Mithal, Dr Anita Mitra, Ms Gillian E Mobb, Mr Leslie E F Moffat, Mr Mokete, Dr Julian Money-Kyrle, Mr Bruce Montgomery, Mr Martin P Moody, Mr Roland Morley, Mr Sean B Morris, Professor Patrick Morrison, Dr Diana Mort, Mr Amir H Mostafid, Mr Hanif Motiwala, Mr Gulzar Mufti, Mr Gordon Muir, Mr Faiz Mumtaz, Mr Michael Murphy, Mr Keith W Murray, Dr Alexandra Murray, Dr Shirley Murrell, Dr D Muthukumar, Mr Harry Naerger, Mr Siva Namasivayam, Mr Vinod Nargund, Mr Nawrocki, Mr Donald Neilson, Dr A Nethersell, Mr Julian Barwell, Dr Jacqueline C Newby, Dr Hugh Newman, Dr R Newton, Mr Neil Oakley, Mr P J O'Boyle, Mr J O'Brien, Mr Tim S O'Brien, Dr H O'Donnell, Mr Neil O'Donoghue, Mr E O'Donoghue, Mr Chris Ogden, Mr Hemant Ohja, Professor Tim Oliver, Mr Eng K Ong, Mr P O'Reilly, Dr J S O'Rourke, Mr David Osborn, Dr Peter Ostler, Professor Joe O'Sullivan, Dr J Owen, Mr Edward Palfrey, Dr Miguel Panades, Dr Niki Panakis, Mr M Pancharatnam, Mr Michalakis L Pantelides, Dr U Panwar, Dr Omi Parikh, Dr Chris Parker, Mr Christopher H Parker, Mr Bohdan T Parys, Dr Sarah Pascoe, Mr Anup Patel, Dr Joan Paterson, Mr S Pathack, Ms Jhumur Pati, Dr Helen Patterson, Dr Pattu, Mr A Paul, Dr Heather Payne, Dr David Peake, Dr I Pedley, Mr A Pengelly, Mr Amjad M Peracha, Dr Matthew Perry, Mr Raj Persad, Mr John Peters, Mr N H Philp, Mr T Philp, Dr Lisa M Pickering, Dr Katharine Pigott, Mr R Plail, Dr P Nicholas Plowman, Mr Richard D Pocock, Mr A J Pope, Mr Rick Popert, Mr Tim Porter, Mr John M Potter, Mr Christopher Powell, Dr Thomas B Powles, Mr Krishna Prasad, Mr Seshadri Sri Prasad, Mr J W Prejbisz, Mr Stephen Prescott, Dr Andrew Protheroe, Mr Khaver N Qureshi, Dr Nigel Raby, Dr Narasimhan Ragavan, Mr Palaniappa G S Raju, Dr Prakash B Ramachandra, Dr R Raman, Mr Abhay Rane, Dr Julia Rankin, Mr Y Rao, Mr Hari L Ratan, Mr Ramachandran Ravi, Dr K Ravishankar, Dr Read, Mr Paul J Reddy, Mr Peter R Rimington, Dr Peter A Ritchie, Dr J Trevor Roberts, Mr Andrew Robertson, Dr Angus Robinson, Dr Anne C Robinson, Mr Lee Q Robinson, Mr Mark A Rochester, Mr P B Rogers, Mr Tomas P Rosenbaum, Mr Neil Rothwell, Mr Carl Rowbotham, Mr Rowe, Dr Kathryn Rowley, Dr Deborah Ruddy, Mr John Rundle, Dr John M Russell, Mr P G Ryan, Dr A Sabharwal, Dr Anand K Saggar, Dr Ali Samanci, Mr Vijay K Sangar, Mr M F Saxby, Mr Hartwig Schwaibold, Dr John E Scoble, Dr Christopher Scrase, Mr Selim, Mr Henry Sells, Mr Krishna K Sethia, Mr David C Shackley, Dr Shaffer, Dr Nihil Shah, Dr D Shakespeare, Dr Sue Shanley, Mr Neerah K Sharma, Dr Denise J Sheehan, Dr Elizabeth Sherwin, Dr Poh Lin Shum, Dr LucySide, Dr Norma Sidek, Professor Karol Sikora, Dr R Simcock, Mr Andrew M Sinclair, Mr Pravin Singh, Dr M Siva, Mr Michael F Smith, Mr James Smith, Dr Michael Sokal, Mr Graham M Sole, Mr Mark J Speakman, Dr Alexander Spiers, Dr Thiagarajan Sreenivasan, Dr Narayanan N Srihari, Mr Srinivasan, Mr Rajagopalan Sriram, Dr John N Staffurth, Dr D Stewart, Dr Andrew Stockdale, Mr Mark A Stott, Mr M J Stower, Mr John R Strachan, Professor Nicholas S A Stuart, Dr Elaine Sugden, Mr Duncan Summerton, Dr Santhanam Sundar, Mr S K Sundaram, Mr Gokarakonda Suresh, Mr Shabbir Susnerwala, Mr Kuchibhotla S Swami, Miss Stephanie J Symons, Dr Isabel Syndikus, Dr Saad Tahir, Dr J Tanquay, Dr John W Taylor, Dr J W Taylor, Mr T Terry, Dr Robert J Thomas, Mr Stephen A Thomas, Mr Alan Thompson, Dr Alastair H Thomson, Dr A Thurston, Dr Owen Tilsley, Mr Stuart F Tindall, Dr K Tipples, Dr Tong, Mr Hamid Toussi, Dr Elizabeth W Toy, Professor Richard C Trembath, Mr David N Tulloch, Mr Kevin J Turner, Mr James Tweedle, Dr C J Tyrell, Mr N Umez-Eronini, Mr Graeme H Urwin, Mr Justin A Vale, Dr Van As, Dr Nicholas Van As, Dr Subramaniam Vasanthan, Mr Sean Vesey, Dr Maria Vilarino-Varela, Dr John Violet, Mr Jaspal Virdi, Dr Robert Wade, Dr Katherine Waite, Mr E M Walker, Mr Roger Walker, Mr David M A Wallace, Mr Nicholas A Watkin, Mr M E Watson, Professor J H Waxman, Mr Brian Waymont, Dr Andrew Weaver, Mr Ralph J Webb, Mr Andrew Wedderburn, Dr Paula Wells, Mr G D Wemyss-Holden, Mr P M T Weston, Dr Duncan Wheatley, Mr P Whelan, Dr D Whillis, Mr Adam D Wilde, Dr Vicki Wiles, Dr Marie Wilkins, Mr John H Williams, Mr Simon Williams, Mr Michael Willis, Mr Michael I Wills, Mr Richard Wilson, Mr J R Wilson, Mr Mathias H Winkler, Dr Marcus Wise, Mr Simon Woodhams, Professor C Woodhouse, Dr Cathryn Woodward, Dr Woolf, Mr K A Woolfenden, Dr Jane Worlding, Mr Mark Wright, Dr WYLIE, Dr James P Wylie, Dr Chris Wynne, Ms Angelika Zang, Dr A Zarkar,

**The UK ProtecT Study Collaborators**

Angela Cox**,** Paul M. Brown**,** Anne George**,** Gemma Marsden**,** Athene Lane**,** Michael DavisPrasad Bollina, Sue Bonnington, Lynne Bradshaw, James Catto, Debbie Cooper, Liz Down, Andrew Doble, Alan Doherty, Garrett Durkan, Emma Elliott, David Gillatt, Pippa Herbert, Peter Holding, Joanne Howson, Mandy Jones, Roger Kockelbergh, Rajeev Kumar, Howard Kynaston, Athene Lane, Teresa Lennon, Norma Lyons, Hing Leung, Malcolm Mason, Hilary Moody, Philip Powell, Alan Paul, Stephen Prescott, Derek Rosario, Patricia O'Sullivan, Pauline Thompson, Sarah Tidball.

**Members of the PRACTICAL consortium by studies who are not listed as authors**

The Institute of Cancer Research & The Royal Marsden NHS Foundation Trust

Cyril Fisher, Charles Jameson

AUSTRALIA

Melbourne

Melissa C. Southey, John L. Hopper, Dallas R. English**,** John Pedersen

Queensland

Srilakshmi Srinivasan, Felicity Lose, Amanda Spurdle

Australian Prostate Cancer BioResource: Gail Risbridger, Wayne Tilley, Lisa Horvarth

Australian Prostate Cancer Bio Resource-QLD node: Peter Heathcote, Glenn Wood, Greg Malone, Hema Samaratunga, Pamela Saunders, Allison Eckert, Trina Yeadon, Kris Kerr, Angus Collins, Megan Turner

BULGARIA

PCMUS study

Medical University, Sofia,Department of Urology: Chavdar Slavov, Vanio Mitev, Elenko Popov

Molecular Medicine Center and Department of Chemistry and Biochemistry: Darina Kachakova, Atanaska Mitkova, Teodora Goranova, Gergana Stancheva, Olga Beltcheva, Rumyana Dodova

Department of General and Clinical Pathology: Aleksandrina Vlahova, Tihomir Dikov, Svetlana Christova

DENMARK

CPCS1

Department of Urology, Herlev Hospital, Copenhagen University Hospital,

Herlev, Denmark: Peter Klarskov, Børge G. Nordestgaard, M. Andreas Røder, Sune F. Nielsen, Stig E. Bojesen

CPCS2

Department of Urology, Rigshospitalet, Copenhagen University Hospital, Copenhagen, Denmark: Prof. DMSc Peter Iversen

FINLAND

University of Tampere and Tampere University Hospital, Tampere, Finland: Tiina Wahlfors, Teuvo LJ Tammela,Anssi Auvinen

The Finnish Cancer Registry, Helsinki, Finland: Liisa Määttänen

GERMANY

ESTHER

Saarland Cancer Registry, Saarbrücken, Germany: Christa Stegmaier

German Cancer Research Center (DKFZ), Heidelberg, Germany: Aida Karina Dieffenbach, Dietrich Rothenbacher

Ulm

Department of Urology, University Hospital Ulm, Germany: Walter Vogl, Antje E. Rinckleb**,** Manuel Luedeke**,** Mark Schrader

Institute of Human Genetics, University Hospital Ulm, Germany: Josef Hoegel

Department of Urology, Technical University Munich, Germany: Kathleen Herkommer

POLAND

Dominika Wokolorczyk, Jan Lubinski

PORTUGAL

IPO-Porto Study

Department of Pathology, Portuguese Oncology Institute, Porto, Portugal: Rui Henrique

Department of Genetics, Portuguese Oncology Institute, Porto, Portugal: Carmen Jerónimo, Pedro Pinto, Joana Santos, João D. Barros-Silva, Sofia Maia, Paula Paulo

SWEDEN

CAPS

Jan Adolfsson, Pär Stattin, Jan-Erik Johansson

STHM1

Carin Cavalli-Björkman, Ami Rönnberg Karlsson, Michael Broms

UNITED KINGDOM

The Institute of Cancer Research & The Royal Marsden NHS Foundation Trust, London

Cyril Fisher, Charles Jameson

EPIC-BPC3

Department of Epidemiology and Biostatistics, School of Public Health, Imperial College, London, United Kingdom: Hans Wallinder, Sven Gustafsson

UNITED STATES OF AMERICA

FHCRC

Fred Hutchinson Cancer Research Center, Seattle, US: Suzanne Kolb, Danielle M. Karyadi

MAYO

Shannon K McDonnell**,** Lori Tillmans**,** Shaun Riska

MEC-BPC3

Department of Preventive Medicine, Keck School of Medicine, University of Southern California, Los Angeles, CA, USA: Mariana C Stern, Roman Corral, Amit D. Joshi, Ahva Shahabi, Dan Stram

Epidemiology Program, University of Hawaii Cancer Center, Department of Medicine, John A. Burns School of Medicine, Univerisity of Hawaii, Honolulu, HI, USA: Kolonel Laurence

MOFFIT

Thomas A. Sellers, Hui-Yi Lin, Julio Pow-Sang, Hyun Y. Park, Selina Radlein, Maria Rincon

James A Haley VA Hospital, Tampa, FL, USA: Babu Zachariah

SALT LAKE CITY, UTAH

Robert A Stephenson, Craig Teerlink
