# Supplementary material for: Fine-Mapping the HOXB Region Detects Common Variants Tagging a Rare Coding Allele: Evidence for Synthetic Association in Prostate Cancer
Source: PLoS Genet. 2014 Feb 13;10(2):e1004129. doi: 10.1371/journal.pgen.1004129 (PMC3923678; doi:10.1371/journal.pgen.1004129)
Supplement: Supplementary Information S2 — Details of additional funding, abbreviations and URLs. (DOCX) [file pgen.1004129.s004.docx]

**Supplementary Information S2**

**Details of additional funding, abbreviations and URLs.**

**Further Funding details**

The FHCRC, Mayo, MCCS, Tampere, UKGPCS and Ulm groups are part of the ICPCG supported by NIH Grant No. U01 CA089600-04.

The Prostate Cancer Program of Cancer Council Victoria also acknowledge grant support from The National Health and Medical Research Council, Australia (126402, 209057, 251533, 396414, 450104, 504700, 504702, 504715, 623204, 940394, 614296), VicHealth, Cancer Council Victoria, The Prostate Cancer Foundation of Australia, The Whitten Foundation, PricewaterhouseCoopers, and Tattersall’s. EAO, DMK, and EMK acknowledge the Intramural Program of the National Human Genome Research Institute for their support.

The QLD research is supported by The National Health and Medical Research Council, Australia Project Grant [390130, 1009458] and Enabling Grant [614296 to APCB]; the Prostate Cancer Foundation of Australia (Project Grant [PG7] and Research infrastructure grant [to APCB]).

We would like to acknowledge the support of The University of Cambridge, Cancer Research UK. Cancer Research UK grants [C8197/A10123] and [C8197/A10865] supported the genotyping team. We would also like to acknowledge the support of the National Institute for Health Research which funds the Cambridge Bio-medical Research Centre, Cambridge, UK. We would also like to acknowledge the support of the National Cancer Research Prostate Cancer: Mechanisms of Progression and Treatment (PROMPT) collaborative (grant code G0500966/75466) which has funded tissue and urine collections in Cambridge.

We are grateful to staff at the Welcome Trust Clinical Research Facility, Addenbrooke’s Clinical Research Centre, Cambridge, UK for their help in conducting the ProtecT study.  We also acknowledge the support of the NIHR Cambridge Biomedical Research Centre, the DOH HTA (ProtecT grant) and the NCRI / MRC (ProMPT grant) for help with the bio-repository. The UK Department of Health funded the ProtecT study through the NIHR Health Technology Assessment Programme (projects 96/20/06, 96/20/99). The ProtecT trial and its linked ProMPT and CAP (Comparison Arm for ProtecT) studies are supported by Department of Health, England; Cancer Research UK grant number C522/A8649, Medical Research Council of England grant number G0500966, ID 75466 and The NCRI, UK. The epidemiological data for ProtecT were generated though funding from the Southwest National Health Service Research and Development. DNA extraction in ProtecT was supported by USA Dept of Defense award W81XWH-04-1-0280, Yorkshire Cancer Research and Cancer Research UK. The authors would like to acknowledge the contribution of all members of the ProtecT study research group. The views and opinions expressed therein are those of the authors and do not necessarily reflect those of the Department of Health of England. The bio-repository from ProtecT is supported by the NCRI (ProMPT) Prostate Cancer Collaborative and the Cambridge BMRC grant from NIHR.

The CGEMS and PLCO studies were funded by the Intramural Research Program of the Division of Cancer Epidemiology and Genetics, National Cancer Institute, NIH.

The Mayo group was supported by the US National Cancer Institute (R01CA72818).

The MEC was support by NIH grants CA63464, CA54281 and CA098758.

The Moffitt group was supported by the US National Cancer Institute (R01CA128813, PI: J.Y. Park).

The USC study was supported by the US National Cancer Institute (R01CA84979) and by the California Cancer Research Program (99-00524V-10258).

The San Francisco Bay Area Prostate Cancer Study was supported by the California Cancer Research Fund (99-00527V-10182).

The Tampere (Finland) study was supported by the Academy of Finland Grant 116437 and 251074, The Finnish Cancer Organisations, Sigrid Juselius Foundation, and The Medical Research Fund of Tampere University Hospital (# 9N069). The PSA screening samples were collected by the Finnish part of ERSPC ([European Study of Screening for Prostate Cancer](http://www.erspc.org/)).

The FHCRC studies were supported by grants RO1 CA056678, RO1 CA082664, and RO1 CA092579 from the National Cancer Institute, National Institutes of Health, with additional support from the Fred Hutchinson Cancer Research Center.

The Department of Medical Epidemiology and Biostatistics, Karolinska Institute, Stockholm, Sweden was supported by the Cancer Risk Prediction Center (CRisP; [www.crispcenter.org](http://www.crispcenter.org/)), a  Linneus Centre (Contract ID 70867902) financed by the Swedish Research Council, Swedish Research Council (grant no K2010-70X-20430-04-3), the Swedish Cancer Foundation (grant no 09-0677), the Hedlund Foundation, the Söderberg Foundation, the Enqvist Foundation, ALF funds from the Stockholm County Council. Stiftelsen Johanna Hagstrand och Sigfrid Linnér’s Minne, Karlsson’s Fund for urological and surgical research. We thank and acknowledge all of the participants in the Stockholm-1 study. We thank Carin Cavalli-Björkman and Ami Rönnberg Karlsson for their dedicated work in the collection of data. Michael Broms is acknowledged for his skilful work with the databases. KI Biobank is acknowledged for handling the samples and for DNA extraction. Hans Wallinder at Aleris Medilab and Sven Gustafsson at Karolinska University Laboratory are thanked for their good cooperation in providing historical laboratory results.

The PCMUS study was supported by the Bulgarian National Science Fund, Ministry of Education, Youth and Science (contract DOO-119/2009) with additional support from the Science Fund of Medical University - Sofia (contract 51/2009; 8I/2009).

SCCS is funded by NIH grant R01 CA092447, and SCCS sample preparation was conducted at the Epidemiology Biospecimen Core Lab that is supported in part by the Vanderbilt-Ingram Cancer Center (P30 CA68485). Data on SCCS cancer cases used in this publication were provided by the Alabama Statewide Cancer Registry; Kentucky Cancer Registry, Lexington, KY; Tennessee Department of Health, Office of Cancer Surveillance; Florida Cancer Data System; North Carolina Central Cancer Registry, North Carolina Division of Public Health; Georgia Comprehensive Cancer Registry; Louisiana Tumor Registry; Mississippi Cancer Registry; South Carolina Central Cancer Registry; Virginia Department of Health, Virginia Cancer Registry; Arkansas Department of Health, Cancer Registry, 4815 W. Markham, Little Rock, AR 72205. The Arkansas Central Cancer Registry is fully funded by a grant from National Program of Cancer Registries, Centers for Disease Control and Prevention (CDC). Data on SCCS cancer cases from Mississippi were collected by the Mississippi Cancer Registry which participates in the National Program of Cancer Registries (NPCR) of the Centers for Disease Control and Prevention (CDC). The contents of this publication are solely the responsibility of the authors and do not necessarily represent the official views of the CDC or the Mississippi Cancer Registry.

The Keith and Susan Warshaw Fund, C. S. Watkins Urologic Cancer Fund and The Tennity Family Fund supported the Utah study. The project was supported by Award Number P30CA042014 from the National Cancer Institute.

The ESTHER study was supported by a grant from the Baden-Württemberg Ministry of Science, Research and Arts.

The IPO-Porto study was in part supported by Liga Portuguesa Contra o Cancro and Fundação para a Ciência e a Tecnologia.

John Hopper is an Australia Fellow of the NHMRC. A. Spurdle is an NHMRC Senior Research Fellow, J. Clements is an NHMRC Principal Research Fellow, and J. Batra is an NHMRC Training Fellow. QLD study is currently supported by NHMRC grants APP1009458 and APP1050742.

**Abbreviations**

COGS – Collaborative Oncology Gene-Environment Study

GWAS – Genome-wide Association Study

MAF – Minor Allele Frequency

PrCa – Prostate Cancer

SNP – Single Nucleotide Polymorphism

**URLs**

<http://www.cancerresearchuk.org/cancer-info/cancerstats/types/prostate/incidence/>

[www.icr.ac.uk/ukgpcs](http://www.icr.ac.uk/ukgpcs)

<http://ec.europa.eu/research/health/medical-research/cancer/fp7-projects/cogs_en.html>

<http://ccge.medschl.cam.ac.uk/consortia/practical>

<http://mathgen.stats.ox.ac.uk/impute/data_download_1000G_phase1_integrated.html>

<http://pngu.mgh.harvard.edu/purcell/plink/>
